# Evaluating the Effectiveness of African School of Hypertension for Non-Physician Health Workers, a Qualitative Study: QuASH Hypertension Study

**DOI:** 10.5334/gh.1343

**Published:** 2024-07-31

**Authors:** Godsent C. Isiguzo, Oluseyi A. Adejumo, Ifeanyi E. Nwude, Uzochukwu M. Amaechi, Ayodele Y. Ayoola, Manmak H. Mamven, Reuben K. Mutagaywa, Ayodipupo S. Oguntade, Kelechi G. Isiguzo, Abiodun M. Adeoye, Beheiry M. Hind, Alfred Doku, Albertino A. Damasceno, Lucia D. Mbulaje, Sebastian C. Marwa, Akinyemi Aje, Louis Avorkliya, Lamin E. S. Jaiteh, Florence K. Akumiah, Elijah N. Ogola, Tangeni Auala, Chinonso J. Okereke, Basden J. Onwubere, Abiodun A. Akintunde, Augustine N. Odili

**Affiliations:** 1 Division of Cardiology, Department of Internal Medicine Alex Ekwueme Federal University Teaching Hospital and Ebonyi State University, Abakaliki Ebonyi State, Nigeria; 2 Department of Internal Medicine University of Medical Sciences, Ondo State, Nigeria; 3 Department of Business Development and Communication, Society for Public Health, and Social Development, Lugbe, Abuja, Nigeria; 4 Department of Human Resources, Society for Public Health, and Social Development, Lugbe, Abuja, Nigeria; 5 Federal Teaching Hospital Gombe/Gombe State University, Nigeria; 6 Department of Medicine University of Abuja, Nigeria; 7 Muhimbili Orthopedic Institute, Tanzania; 8 Clinical Trial Service Unit and Epidemiological Studies Unit (CTSU), Big Data Institute, Oxford Population Health, University of Oxford, United Kingdom; 9 Department of Early Childhood Education University of Pretoria, South Africa; 10 Cardiovascular Genomic Unit Institute of Cardiovascular Diseases, College of Medicine University of Ibadan, Ibadan, Nigeria; 11 Department of Physiology, Director of Educational Development and Research Centre, Faculty of Medicine, International University of Africa, Sudan; 12 University of Ghana Medical School, Korle-Bu Teaching Hospital, Accra, Ghana; 13 Faculty of Medicine, Eduardo Mondlane University, Maputo, Mozambique; 14 Queen Elizabeth Central Hospital, Blantyre, Malawi; 15 Muhimbili University of Health and Allied Sciences, Dar es Salaam, Tanzania; 16 Department of Medicine, University College Hospital, Queen Elizabeth Road, Ibadan, Nigeria; 17 Dept of Medicine and therapeutics, Korle Bu Teaching Hospital, Korle Bu, Accra, Ghana; 18 Department of Internal Medicine, Edward Francis Small Teaching Hospital/School of Medicine & Allied Health Sciences, University of the Gambia Banjul, Gambia; 19 National Cardiothoracic Centre, Korle-Bu, Accra, Ghana; 20 Department of Clinical Medicine and Therapeutics, University of Nairobi, Nairobi, Kenya; 21 Division of Adult Cardiology, Windhoek Central Hospital Complex, Windhoek, Republic of Namibia; 22 Department of Medicine and Surgery, University of Abuja, Abuja, Nigeria; 23 University of Nigeria Teaching Hospital, Enugu, Nigeria; 24 Department of Medicine, Ladoke Akintola University of Technology, Ogbomoso, Nigeria; 25 Department of Internal Medicine, Faculty of Clinical Sciences, College of Health Sciences, Main Campus, University of Abuja, Abuja, Nigeria

**Keywords:** African Hypertension School, task sharing, task shifting, feedback

## Abstract

**Background:**

The implementation of task sharing and shifting (TSTS) policy as a way of addressing the shortage of physicians and reducing the burden of hypertension in Africa birthed the idea of the African School of Hypertension (ASH). The ASH is saddled with the responsibility of training non-physician health workers across Africa continent in the management of uncomplicated hypertension.

**Aim:**

To get feedback from some faculty members and students who participated in the first ASH programme.

**Methods:**

This was a cross-sectional exploratory qualitative study conducted among eight students and eight faculty members. Feedback from the program was obtained by conducting in-depth interviews centred on description of course content; expectations and knowledge acquired from ASH; level of interaction between students and faculty members; challenges faced during the ASH; level of implementation of acquired training; and suggestions to improve subsequent ASH programs

**Results:**

The course content of the ASH was described as simple, appropriate and adequate while interaction between students and faculty members were highly cordial and engaging. New knowledge about hypertension management was acquired by the students with different levels of implementation post-graduation. Some identified challenges with the ASH program were poor internet connectivity during lectures, non-uniformity of TSTS policies and hypertension management guidelines across Africa, technical problems with hypertension management app and low participation from other African countries apart from Nigeria. Some recommendations to improve ASH program were development of a uniform hypertension management guideline for Africans, wider publicity of the ASH, interpretation of lectures into French and Portuguese languages and improvement of internet connectivity.

**Conclusion:**

The ASH programme has largely achieved its objectives with the very encouraging feedback received from both faculty members and the students. Steps should be taken to address the identified challenges and implement the suggested recommendations in subsequent ASH program to sustain this success.

## Introduction

Non-communicable diseases (NCDs) are a leading cause of death globally, accounting for 41 million deaths yearly (1). The burden of NCDs is enormous, especially in low- and middle-income countries (LMICs) compared to high-income countries (HICs) (1). The rising incidence of some cardiovascular risk factors such as hypertension, diabetes mellitus, dyslipidaemia, physical inactivity, obesity, and air pollution in LMICs account for this higher burden of NCDs (1).

Hypertension, a major driver of NCDs has been on the rise globally. The number of people with hypertension doubled to over a billion between 1990 and 2019 (2). The burden of hypertension is also higher in the LMICs with 88% of hypertension-related global deaths occurring in these countries (3). One of the effective strategies proven to reduce the burden of NCDs is early diagnosis and optimal control of hypertension. Surprisingly, there is low awareness of hypertension in the majority of those who have hypertension (4,5). Also, despite the existence of effective hypertensive medications, a high proportion of those with hypertension who are on treatment have suboptimal blood pressure control (4,5). Factors associated with suboptimal BP control include physicians’ inertia to optimize antihypertensive medications, non-adherence with medications, and poor health literacy (6). There is a need for better management of hypertension because suboptimal blood pressure control is associated with a higher prevalence of major cardiovascular events and higher financial and health costs (7).

A team-based approach has been recommended by the Centre for Disease Control in the diagnosis and better management of hypertension at the primary level in communities (8). This involves task sharing with non-physician healthcare workers such as nurses and community health extension workers in the diagnosis and management of uncomplicated hypertension. The World Health Organization also supports and recommends task shifting and sharing (TSTS) in the management of adults with hypertension especially in LMICs with a limited number of physicians (9,10). Task shifting occurs when a task is transferred or delegated while task sharing occurs when tasks are completed collaboratively between providers with different levels of training (11).

Task shifting and sharing has been successfully employed in the management of communicable diseases such as HIV and provision of maternal and child health in various parts of Africa with tremendous success (12,13). The evidences that support the effectiveness of TSTS in the management of hypertension were analysed by Anand et al. (14) and Ogungbe et al. (15). They observed a significant reduction in blood pressure of those with hypertension managed by non-physician health workers like pharmacists, nurses, dieticians, and community health extension workers. Significant reduction in blood pressure among those with hypertension has also been reported at non-physician-led clinics in several studies (16,17,18).

Training of non-physician health workers is one of the major factors that determine the successful implementation of TSTS in the management of diseases. This is supported by previous reports that showed a positive association between the amount of blood pressure reduction and the level of clinical training among non-physician health workers involved in hypertension management using TSTS (14,15). Aifah et al. (19) also reported the need for training of non-physician health workers for successful implementation of TSTS in hypertension management.

The need to implement TSTS policy as a way of addressing the shortage of physicians and reducing the burden of hypertension in Africa birthed the idea of the African School of Hypertension (ASH) which was saddled with the responsibility of training non-physician health workers and non-health workers across Africa in the management of uncomplicated hypertension (20). It was set up by the ISH Africa Regional Advisory Group (RAG) and the first ASH program held between August 2022 and January 2023 (20).

This **QU**alitative study on the effectiveness of the **A**frican **S**chool of **H**ypertension (ASH) initiative (QuASH hypertension study) assessed the first ASH programme by getting feedback from the faculty members and students who participated in the programme. This qualitative evaluation was designed to build ownership of the ASH strategy among the stakeholders, aid in a deeper understanding of TSTS in hypertension management, and provide the framework for future courses in ASH. The QuASH study is expected to generate novel and context-specific insights into hypertension care in Africa, which can then be used to inform national planning for the healthcare workforce.

## Method

### Study design

This study was a cross-sectional exploratory qualitative study that involved both ASH students and faculty members. It was conducted over a four-week period in April, 2023. In-depth interview based on pre-selected questions were conducted virtually among the participants, three months after the completion of first ASH program. All in-depth interview (IDI) sessions were audio-recorded and transcribed.

### Study setting

The ASH was set up by the ISH Africa RAG to deepen the understanding of hypertension among non-physician health workers and improve hypertension control in the rural areas in Africa (20). The training duration was a total of 20 weeks and scheduled in two phases; online lecture phase (scheduled for 8 weeks) and a mentorship phase (scheduled for 12 weeks). The ASH trained a total of 94 non-physician health workers from 8 African countries.

### Mode of assessment in the ASH

The faculty members were asked to set multiple choice questions to cover topics took by them (Supplementary Table). These questions were then submitted to the Director of ASH for review and final collation. The students had a pretest evaluation before commencement of lectures to assess their level of knowledge. They also had revisions on previously taken lectures and assessment in the form of quizzes during the course of the program to evaluate the comprehension of the courses they were taught. At the end of the program, they had a comprehensive assessment that covered all the topics they were taught. The students were also asked to provide feedback on the contents and delivery of the lectures.

### Mentorship and supervision of students using Oyoyo App

The students had 12 weeks of direct supportive supervision of their field work after they had completed their lectures. They were encouraged to implement the acquired knowledge in their communities under the supportive supervision and mentorship of assigned mentors. The implementation of acquired knowledge and supervision was done using the Oyoyo App.

The Oyoyo App is a purpose built application that runs on android and iPhone Operating System for real time monitoring of the non-physician health workers’ activities as part of the supportive supervision by the in-country mentors. The non-physician health workers enter clients’ bio-data, blood pressure measurement and prescription into the app. The physician mentor reviews all the information entered by all his/her mentees in real time and approves or advises a change in the management plan as the case may be. The app also enables data collection from all the sites where non-physician health workers are operating.

### Establishment of the QuASH hypertension cohort

In the formative stage of QuASH hypertension study, the qualitative team had deliberations on the selection of relevant stakeholders to be interviewed and the contents of in-depth interview which were informed by feedback from interactive sessions with the students and the objective of this study. The interview questions were subsequently sent to the faculty board of ASH for expert review. The questions were then standardized by pretesting the questions, evaluating the responses obtained from the interview and modifying the questions wherever necessary.

**Figure 1:**
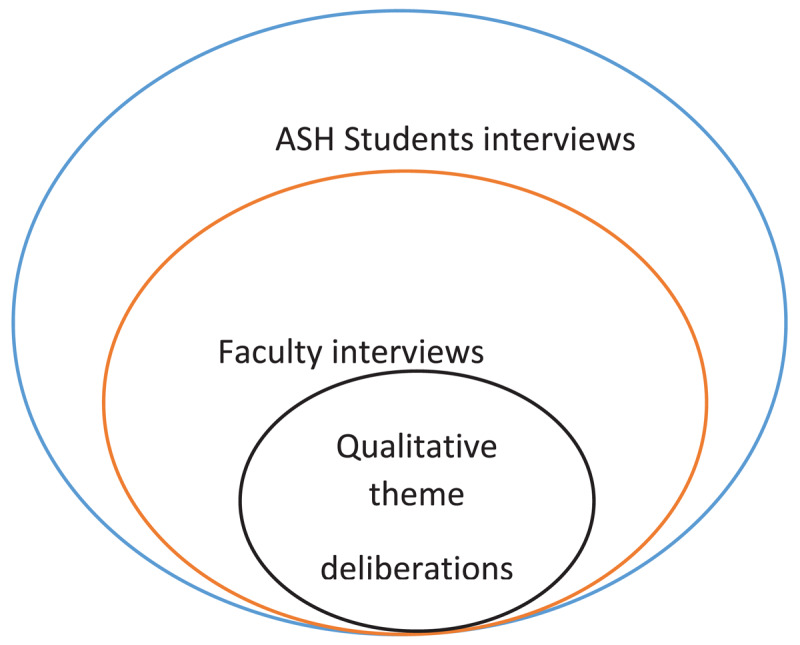
QuASH hypertension formative concept.

The QuASH hypertension study participants consisted of eight ASH fellows drawn from the 94 inaugural students and eight faculty members (Figure 1). The participants were purposively selected.

### Data collection

The interview was conducted by asking open-ended questions accompanied by follow-up probes. The main concept areas evaluated among the students were prior knowledge of hypertension; level of experience with managing patients with hypertension; contents of curriculum; new information gained in the course; challenges encountered during participation in the programme; level of implementation of acquired knowledge after graduation; challenges faced during implementation of acquired knowledge; and recommendations for improvement of future ASH programmes. The interview questions for the faculty members were centered on the description of quality of the programme curriculum; level of interaction between them and the students; mentorship experience with the ASH fellows; challenges faced during the program; and recommendations for improvement of future ASH programmes.

### Qualitative Data Processing and Analysis Procedure

The audio recordings were transcribed using the transcription template for the study. The transcripts were reviewed by another group of researchers and transferred into ATLAS.ti version 9.0.22.0 to organize and sort for further data analysis (coding). Two researchers read the transcripts for familiarization and codebook development.

The codebook was developed using a hybrid of inductive approach, i.e., drawing from the qualitative data in the transcripts, and deductive approach, i.e., drawing from the interview guide. The researchers coded the same transcripts to ensure that the codes were consistently applied if need be.

Discrepancies were resolved through a consensus-building approach guided by the research objectives. Thereafter, ATLAS.ti projects from different researchers were carefully reviewed through the inter-coder reliability tool and later merged. The quotations were pulled together to understand patterns across the data. The analysis was guided by the study objective which was used to evaluate the first ASH programme. The data analysis adopted a thematic and content analytical approach which was used to derive recurring categories and themes from the data.

### Ethical consideration

This study was conducted in accordance to the Declaration of Helsinki, a statement of ethical principles for medical research involving human subjects. The study obtained ethical approval from the Human Research Ethics Committee of University of Abuja with approval number UATH/HREC/PR/202403150. All participants provided written informed consent for the study and their data were de-identified and entered into a secure password-protected database.

## Results

Eight students and eight faculty members from Nigeria, Ghana, Sudan and Tanzania participated in the key informant interviews. The themes and subthemes generated are presented in Table 1.

**Table 1:** Themes and Subthemes.


S/N	THEMES	SUBTHEMES

1	Students’ knowledge before attending ASH programme	Lack of knowledge of definition of hypertension Lack of knowledge of measurement and classification of blood pressure Lack of knowledge of hypertension management Some knowledge of lifestyle modification in hypertension

2	Description of course content of ASH programme	Simple Appropriate Adequate

3	Expectations and New knowledge gained from ASH programme	Fulfilled Expectations Blood pressure classification and measurement Hypertension in children Hypertension complications Hypertension diagnosis and management

4	Interaction between student and faculty members during ASH programme	Highly interactive Regular feedback Well engaging

5	Challenges experienced during ASH programme	Poor internet connectivity Differences in hypertension management protocol and task shifting polices in different countries Technical problems with hypertension management App Low participation from other non-Nigerian African countries

6	Level of implementation of acquired training during ASH programme	Regular home visit Activation of patients’ management on App Diagnosis and prompt referral

7	Challenges faced with implementation of training acquired during ASH programme	Difficulty with App operation Non-adherence of patients to treatment Limitation of roles by national policies Delay feedback from mentors

8	Recommendations for improvement of ASH programme	Use of more audiovisual Improvement in internet connectivity Well defined criteria for recruitment of students Development of uniform hypertension treatment protocol for Africans Use of interpreters to present lectures in French Wide publicity of hypertension school


### Theme 1: Students’ knowledge before attending ASH programme

It was observed that the students had limited knowledge about hypertension, especially in the areas such as hypertension definition, blood pressure measurement, blood pressure classification, and hypertension treatment. However, some students had good knowledge of lifestyle modification in hypertension.

*I have a little information about hypertension before the course*. (Student from Sudan)

*I know about lifestyle modification*. (Student from Tanzania)

### Theme 2: Description of course content of ASH programme

The faculty members described the course content of the program to be appropriate, simple, and adequate.

*It was direct and simple enough for anyone in the health sector to understand and implement in their practices*. (Faculty member from Nigeria)

*The focus of this training or course was for non-physicians. The contents were targeting these people. They were given the basics of hypertension from the definition, prevalence, management, complications and diagnosis. I think the content was really focused and well planned. The content was really adequate*. (Faculty member from Tanzania)

### Theme 3: Expectations and new knowledge gained from ASH programme

The students’ expectations of attending ASH were met. There was knowledge acquisition in the areas of blood pressure measurement and classification; hypertension in children; hypertension diagnosis; complications; and hypertension management.

*Specifically gained knowledge was on the classification of hypertension*. (Student from Sudan)

*Before training, I did not know that children can develop hypertension, but during training, I learnt they can develop hypertension*. (Student from Nigeria)

*I got new knowledge on the different blood pressure measuring machines during the lectures. We do not have such machines here in Tanzania; so when I see those machines in other places, I will be able to use them*. (Student from Tanzania)

*My expectation was to get more knowledge on hypertension which I have already gotten*. (Student from Tanzania)

### Theme 4: Interaction between student and faculty members during ASH programme

The interactions between faculty members and students during the ASH program were described as highly interactive, engaging, and cordial.

*I think the interactions were very engaging and any faculty member would be pleased with the kind of attention the students put in and the kind of questions they asked. Even thereafter the sessions, they kept sending questions. One or two of the students have got across to me in the past month and I know that is how they would have gotten across to other members*. (Faculty member from Nigeria)

*They were very interactive, students were asking questions and I was also asking questions*. (Faculty member from Tanzania)

*We were able to join all these African students like one family, one group, one goal and that was really amazing, it’s happening for the first time, and I think this is really an achievement, good achievement*. (Student from Sudan)

### Theme 5: Challenges experienced during ASH programme

The challenges faced during the course of the ASH programmes were differences in management national guidelines for hypertension and task shifting policies, poor internet connectivity, technical problems with the hypertension management app, and low-participation from other African countries.

*The fact that we are dealing with so many countries with different policies which affect the health sector. The issue of the health workers, their competencies and what they are allowed to do or not to do differs from one country to another, so it becomes it is difficult to say what is obtainable here is obtainable somewhere else. I think that is one major challenge that I noticed*. (Faculty Member from Nigeria)

*In Sudan, the internet connectivity was poor. It was difficult for the students to follow and to use the app*. (Faculty member from Sudan)

*The implementation part, there were a lot of issues with the app even for us as the mentors. We had a lot of challenges when we came on board. I think the app component should be improved on. Sometimes you are logging in and you don’t get access*. (Faculty Member from Ghana)

*In Nigeria, we had enough participants unlike other countries where they really need people to work more*. (Faculty Member from Nigeria)

### Theme 6: Implementation of acquired knowledge during ASH programme

Some of the students have started implementing the acquired knowledge in the ASH. This includes management of patients using the app, regular home visits for blood pressure monitoring, diagnosis, and prompt referral of patients with hypertension.

*I have seen about two or three people having high blood pressure and I made a diagnosis and referred them to the nearby center for continuous monitoring*. (Student from Nigeria)

*Yes, I am using the knowledge. I am working at a center and I go home visiting. I have helped a lot of people especially those staying at homes*. (Student from Tanzania)

*Yes, I have already started using the app and I have a lot of patients right now on my Oyoyo App. They have been coming for follow up and I have seen a significant change in their blood pressure*. (Student from Nigeria)

### Theme 7: Challenges faced with the implementation of training acquired knowledge during ASH programme

The challenges encountered by some of the students during implementation of what they learnt were technical challenges with the hypertension management app, non-adherence to medications, delayed feedback from supervising physician, and limitation of task shifting according to the prevailing policies in their country.

*I was fiddling with the Oyoyo app, at a point I had difficulty recording subsequent readings. This morning, I was able to do it*. (Student from Nigeria)

*Challenges I see is that a lot of patients are tired of using their medications every day. I try to put a lot of effort to educate and teach them the importance of the medicines and to give hope. That is the big challenge for me*. (Student from Tanzania)

*Not all the knowledge learnt could be implemented because there we have limited roles*. (Student from Sudan)

*I have even created a WhatsApp group that will help in communicating with our mentors, so that if there are any immediate changes or any clarification, we’ll now communicate within ourselves, but I did not get most of the cooperation*. (Student from Nigeria)

### Theme 8: Recommendations for improvement of ASH programme

Both faculty members and students provided suggestions on ways to improve the ASH program to achieve its objectives. These included improving course content on aspects of non-pharmacological treatment of hypertension, improving methods of lecture delivery by using more audiovisuals, resolving the technical problems with the hypertension management app, and increasing the allotted time to students’ practical sessions.

*The non-pharmacological part should be emphasized so that we can actually look at specific lifestyle interventions and give more details*. (Faculty Member from Ghana)

*The area that I think we need to improve upon is the interaction between the mentors and the students because we have challenges going through the app*. (Faculty Member from Nigeria)

*Try to involve video classes for the students*. (Faculty member from Sudan)

*The area you need to improve is about mentors that are assigned. We don’t know how they are monitoring our apps*. (Student from Nigeria)

*I think we need to put more time, more effort in the application of acquired knowledge*. (Faculty Member from Sudan)

## Discussion

Evaluation of programmes through assessment of feedback from participants is highly valuable in identifying specific areas that require improvement to effectively achieve the set objectives. This study was conducted among some students and faculty members who participated in the maiden ASH programme to assess the effectiveness of the program, identify areas of challenges and seek recommendations from them.

### Course content and delivery evaluation

Both the students and faculty members agreed that the course was comprehensive and adequately reflected the set objectives of the ASH. The mode of delivery was described as simple, easy to understand, and very interactive. The students also confirmed that the faculty members demonstrated competency in teaching various topics during the course of the program. The use of audiovisuals, training manuals, inclusion of fieldwork, more flexible timing, and regular formative and summative assessment were some of the suggestions that were however made to further enrich subsequent ASH programme. The use of audiovisual technology as an instructional method of lecture delivery has been reported to aid students’ comprehension and performance (21).

### New knowledge acquisition and expectations among students

The students attested to the fact that the ASH met most of their expectations and was quite educative to them. Areas of knowledge acquisition include proper definition, recognition, diagnosis, classification, and management of uncomplicated hypertension. Other areas noted by the students are improved knowledge about hypertension in special cases such as in children; early recognition of complications of hypertension; and the complementary roles of non-pharmacological management of hypertension, especially lifestyle modification. This clearly shows that some of the key objectives of the ASH were largely achieved. The implication of this is that graduates of the ASH will have the requisite knowledge and capacity to participate in the management of uncomplicated hypertension with supervision under the TSTS programme.

### Implementation of acquired training and barriers encountered

Few of the students have started implementing the acquired knowledge through the initiation of hypertension screening programme and treatment under the close supervision of their mentors. Others have, however, encountered some barriers in the implementation of the knowledge they have acquired. Some of the identified barriers were technical glitches with the hypertension management app (Oyoyo App), poor compliance with medications among newly diagnosed clients with hypertension who commenced treatment, delayed response from assigned mentors who were to play supervisory roles, and the absence of enabling laws to support active involvement of non-physician health workers in the management of hypertension in some countries. These challenges have prevented the implementation of acquired knowledge for some of the students.

### Encountered challenges during the first edition of ASH

The training was conducted entirely in English language and hence, Portuguese-speaking African countries such as Mozambique, Cape Verde, Guinea-Bissau, Equatorial Guinea, and Angola could not participate due to language barriers, likewise some French-speaking countries too. The consensus was that plans should be made to translate the ASH programmes to other languages, such as Portuguese and French, as it is highly desirable that non-English speaking countries should also be able to participate and benefit from the ASH.

Both faculty members and some students observed that the hypertension treatment guidelines varied across different African countries and therefore, some students had difficulty in using the Oyoyo App, which was developed using hypertension treatment guidelines in Nigeria. These challenges could be solved by building individual country-specific apps that take into consideration the treatment guidelines of specific country or inclusion of unitary components that take care of almost all African countries in the app. This development also highlights the need to harmonize the guidelines and develop a uniform hypertension treatment guideline across all African countries.

The Faculty members and students alluded to the fact that there were several interruptions in the classes due to poor internet connectivity. Poor internet connectivity has been reported to be a major challenge in delivering virtual classes, especially in low and middle-income countries. The inability to download and navigate the Oyoyo App was also noted by both the faculty members and students. This was because of technical hitches arising from the incompatibility of the app with operating systems on their devices and the inability to use the app effectively by individuals who were not quite familiar with its usage. Some of these challenges were however resolved by the technical team before the school programme ended.

### Recommendations

The study recommends the adoption of criteria for admission which prioritizes non-physician health workers. The development of training and instruction methods that utilizes audiovisuals, training manuals, practical field work, and regular formative and summative assessments is also recommended to improve the programme.

To address the identified challenges, mentors should be motivated to ensure more commitment to the supervision of assigned students. There is a need to review existing laws of various African countries to identify any inherent legal limitations on the scope of work/practice of non-physician health workers. This will ensure that the training under the ASH is not futile and that measures are taken to ensure that the activities of non-physician health workers are within the boundaries of legality.

Funding for this program should be provided by regional bodies to complement sustainable development and goals achievement. The funding should include provision of stipends to cover the logistics such as internet expenses for the participants.

Lastly, we also recommend that the ASH programme should be given widespread publicity in order to attract students from across the African region. This will also mean that the language variations across African countries are taken into consideration and that adequate provisions are put in place for the translation of publicity materials and the ASH programmes, itself, in other official languages.

## Conclusion

The ASH programme has largely achieved its objectives with the very encouraging feedback received from both members of the faculty and the students. However, in order to sustain the progress, and to achieve more impact, steps should be taken to address the challenges identified and implement the recommendations provided by both faculty members and students in subsequent ASH programme.

## Data Accessibility Statement

The data that supports the findings of this study are available from the corresponding author upon reasonable request.

## Additional file

The additional file for this article can be found as follows:

10.5334/gh.1343.s1 Supplementary Table. African hypertension school for non-physician health workers course timetable.
