# Supplementary material for: Evaluating the Effectiveness of African School of Hypertension for Non-Physician Health Workers, a Qualitative Study: QuASH Hypertension Study
Source: Glob Heart. 2024 Jul 31;19(1):62. doi: 10.5334/gh.1343 (PMC11295908; doi:10.5334/gh.1343)
Supplement: Supplementary Table. — African hypertension school for non-physician health workers course timetable. [file gh-19-1-1343-s1.pdf]

Supplementary Table

AFRICAN HYPERTENSION SCHOOL FOR NON-PHYSICIAN HEALTH WORKERS COURSE TIMETABLE

Date/Time (WAT)	Lecture Topic	Faculty
Week 1: 3rd September, 2022		
9.00-9.30 a.m	Opening and overview. Introduction to ISH and Allied Health Professionals membership	Odili AN (Nigeria)
9.30am-10.00 am	Pre-course Evaluation	Akintunde AA (Nigeria)
10.00-11:00 a.m.	Introduction and Epidemiology of hypertension	Damasceno A (Mozambique)
Week 2: 10th September, 2022		
9:00-9:45 a.m.	Measurement of BP and diagnosis of hypertension	Onwubere BJC (Nigeria)
9:45-10:30 a.m	Out-of-Office Blood pressure measurement	Isiguzo GC (Nigeria).
10:30-11:30 a.m.	Classification of hypertension	Akintunde AA (Nigeria)
Week 3: 17th September, 2022		
9:00-9:30 a.m	Quiz/Revision of Module 1	All faculties
9:30-10:00 a.m.	Hypertension in Special groups (Diabetes, Elderly.)	Mutagaywa R (Tanzania)
10:00-11:00 a.m.	Hypertension in Special groups (children, pregnancy, HIV)	Ayodipupo S (Nigeria)
Week 4: 24th September, 2022		
9.00-10.00 a.m.	Complications of hypertension, Emergencies and urgencies	Odili AN (Nigeria)
10:00-11:00 a.m.	Cardiovascular Risk Assessment	Odili AN (Nigeria)
Week 5: 1st October, 2022		
9:00 -9:30 a.m.	Quiz/ Revision of Module 2	Akintunde AA (Nigeria)
9:30-10:30 a.m.	Management of hypertension: Lifestyle modifications	Mbulaje L (Malawi)
10.30-11:30 a.m	Management of hypertension: Pharmacologic therapies and side effects	Adeoye A (Nigeria)
Week 6: 8th October, 2022		
9:00- 10:00 am	Hypertension Guidelines and Protocols: Use and challenges	Beheiry H (Sudan)
10.00- 11:00 am	Introduction to Hypertension Management APP	Odili AN (Nigeria)
Week 7: 15th October, 2022		
9:00- 9:30 am	Quiz/Revision Module 3	
9.30-11.00 am	Introduction to hypertension App management 2	Odili AN (Nigeria)
Week 8: 22nd October		
9.00-10.00 am	General Revision	All faculties
10.00-11.00 am	End of Course Assessment	All Faculties
